# Expression of 15-hydroxyprostaglandin dehydrogenase and cyclooxygenase-2 in non-small cell lung cancer: Correlations with angiogenesis and prognosis

**DOI:** 10.3892/ol.2014.2371

**Published:** 2014-07-22

**Authors:** YING LI, SULI LI, DAN SUN, LINLIN SONG, XINMIN LIU

**Affiliations:** 1 Center of Gerontology and Geriatrics, West China Hospital, West China Medical School, Sichuan University, Chengdu, Sichuan 610041, P.R. China; 2 National Key Laboratory of Biotherapy, West China Hospital, West China Medical School, Sichuan University, Chengdu, Sichuan 610041, P.R. China; 3 Department of Geriatrics, Peking University First Hospital, Beijing 100034, P.R. China

**Keywords:** non-small cell lung cancer, 15-hydroxyprostaglandin dehydrogenase, cyclooxygenase-2, angiogenesis, prognosis

## Abstract

The aim of the present study was to investigate the function of 15-hydroxyprostaglandin dehydrogenase (15-PGDH) and cyclooxygenase-2 (COX-2) in angiogenesis and their association with the prognosis of non-small cell lung cancer (NSCLC). Using immunohistochemical staining, the expression of 15-PGDH and COX-2, and the microvessel density (MVD) levels were evaluated in 35 NSCLC specimens. Paracancerous normal lung tissue was collected as control samples from six patients. The correlation of 15-PGDH with COX-2, clinicopathological characteristics, MVD and overall survival (OS) was studied. NSCLC tissues showed a significantly lower expression level of 15-PGDH (P=0.009) and a significantly higher expression level of COX-2 (P=0.004) compared with normal lung tissue. The expression level of 15-PGDH was negatively correlated with MVD (P<0.001) and COX-2 expression (P=0.032). A low expression level of 15-PGDH, a high expression level of COX-2 and high levels of MVD were significantly correlated with a shorter OS time (15-PGDH, P<0.0001; COX-2, P=0.038; MVD, P<0.0001). This study provided clinical evidence that a low expression level of 15-PGDH is associated with a poor prognosis in NSCLC. Furthermore, it was shown that 15-PGDH and COX-2 reciprocally regulate cancer angiogenesis, which may affect the prognosis of patients with NSCLC.

## Introduction

Lung cancer is the most common cause of cancer-related mortality worldwide, for which non-small cell lung cancer (NSCLC) is the most common type of lung cancer. Although there has been a degree of progress in understanding the biology of lung cancer and in the generation of novel therapeutic agents, the five-year overall survival (OS) rate for patients with lung cancer is <15% (1). The low survival rate indicates that novel effective treatment options are required. Identification of molecular pathways involved in lung carcinogenesis may lead to improvements in the management of the disease and the generation of novel-targeted therapies.

Prostaglandin E2 (PGE2) has a predominant function in promoting carcinogenesis and cancer progression, including tumor cell proliferation, invasion, immunosuppression and angiogenesis (2). The key enzymes involved in PGE2 catabolism and the modulation of tumorigenic processes are topics of interest. Cyclooxygenase-2 (COX-2), the rate-limiting enzyme in the synthesis of PGE2, has been regarded as an oncogenesis factor through the accumulation of PGE2 (3). Another central enzyme is 15-hydroxyprostaglandin dehydrogenase (15-PGDH). 15-PGDH oxidizes the 15(*S*)-hydroxyl group of PGE2 to generate 15-keto-prostaglandin, which exhibits greatly reduced biological activity (4). 15-PGDH therefore functions as a COX-2 antagonist in mammalian tissues, including the lung, breast, prostate, placenta and gut (5).

Previous studies have identified the tumor suppressor activity of 15-PGDH in solid tumors. Downregulation of 15-PGDH at the mRNA and protein levels has been found in various malignancies (4,6–10). The genetic ablation of 15-PGDH has been shown to increase the proliferation and motility of human cancer cells (7,9,11). In addition, 15-PGDH overexpression has been observed to lead to the inhibition of tumor angiogenesis by modulating PGE2 and vascular endothelial growth factor expression in NSCLC cells (12). This anti-angiogenic mechanism has a critical function in tumor suppression and provides a novel potential target for anticancer therapy in NSCLC. Various compounds, including COX-2 and histone deacetylation inhibitors, have been identified to induce 15-PGDH as part of their chemo-preventative activities (13,14); however, the clinical studies of 15-PGDH on lung cancer are limited. It is therefore important to explore additional clinical data on the function of 15-PGDH to devise more efficacious treatment strategies for NSCLC.

The present study used an immunohistochemical staining method to analyze the expression of 15-PGDH and COX-2 in NSCLC human specimens and evaluate their clinical prognostic value in patients with NSCLC. Furthermore, the reciprocal regulation of 15-PGDH and COX-2 and their association with angiogenesis was investigated.

## Materials and methods

### Samples from patients

Formalin-fixed, paraffin-embedded tissue specimens from 35 surgically resected primary NSCLC cases, during the period between January 2001 and December 2004, were acquired from the Department of Pathology, Peking University First Hospital (China). These patients did not receive radiotherapy or chemotherapy prior to surgery, and clinical follow up data was available. Paracancerous normal lung tissues from six patients were collected for use as control specimens. The clinical data included age (mean, 60±18 years; range, 41–81 years), gender, smoking history, TNM staging (according to the American Joint Committee on Cancer staging system, 7th edition) (15), histological type and differentiation grading (according to the World Health Organization criteria) (16) and tumor size. All patients were monitored by follow-up until December 2009 where possible. The median follow-up time for survivors was 34 months (range, 3–86 months). The study protocol was approved by the Clinical Research Ethics Committee of the Peking University First Hospital (Beijing, China). Patients provided written informed consent.

### Immunohistochemical staining

Paraffin-embedded tissue blocks for all the cases were consecutively cut into 4-μM sections, deparaffinized in xylene and rehydrated in graded alcohol. Antigen retrieval was performed by boiling the sections for 10 min in a 0.01 M sodium citrate buffer (pH 6.0) in a water bath. Following natural cooling, blocking for endogenous peroxides was performed for 10 min in 3% H_2_O_2_ in methanol. The sections were blocked with 3% bovine serum albumin for 30 min at 37°C and then incubated with rabbit polyclonal antibody directed against human 15-PGDH (1:50 dilution; 160615; Cayman Chemical Co., Ann Arbor, MI, USA), rabbit monoclonal antibody directed against human COX-2 (1:80 dilution; ZA-0515), mouse monoclonal antibody directed against human CD34 (1:100 dilution; ZM-0046) or 0.01 M phosphate-buffered saline (ZLI-9062) (all Zhongshan Golden Bridge Biotechnology Co. Ltd., Beijing, China) for the negative control at 4°C overnight and the next day at 37°C for 1 h. The sections were then incubated with biotinylated goat anti-rabbit immunoglobulin G (SP-9000 Reagent B) for 30 min at 37°C and subsequently incubated with horseradish peroxidase-labeled streptavidin (SP-9000 Reagent C; both Zhongshan Golden Bridge Biotechnology Co. Ltd.) for 20 min at 37°C. Finally, 3,3′-diaminobenzidine (ZLI-9032; Zhongshan Golden Bridge Biotechnology Co. Ltd.) was used as a chromogen and hematoxylin (ZLI-9609; Zhongshan Golden Bridge Biotechnology Co. Ltd.) was used as a counterstain.

### Evaluation of immunohistochemical staining

Evaluation of the stained sections was performed by two pathologists blinded to the clinical information. Each section was scanned at ×100 and ×200 magnification. The expression of 15-PGDH and COX-2 was assessed by determining the immunohistochemistry score (IHS) as follows: IHS = intensity score (absent, 0; weak, 1; moderate, 2; strong, 3) × percentage score (<5%, 0; 5–25%, 1; 25–50%, 2; 50–75%, 3; >75% of total tumor area, 4) (10). The scores ranged from 0 to 12. An IHS of 9–12 was considered strong immunoreactivity, 5–8 was moderate, 1–4 was weak and 0 was negative. For statistical analysis, 15-PGDH and COX-2 scores were divided into a high expression group (strong and moderate immunoreactivity) and a low expression group (weak and negative immunoreactivity) (17).

### Assessment of angiogenesis

An assessment of the microvessel density (MVD) was performed by anti-CD34 staining. This was used to provide an assessment of angiogenesis according to the criteria of Weidner *et al* (18). Tumor sections were scanned at a low magnification (x100) to identify vessels highly positive in CD34 expression, known as ‘hot spots’. The number of CD34-positive cells and cell clusters was counted in five ×200 magnification fields for microvessel counting. The presence of a visible blood vessel lumen was not required for a positive definition. The mean value of the five hot spots was calculated as the MVD for each section. To assess the impact of MVD on prognosis, 35 tumor cases were divided into high and low MVD groups, according to the mean value of the MVD.

### Statistical analysis

χ^2^ or Fisher’s tests were used to compare the statistical differences between the groups. Spearman’s rank test was used to assess the correlation between 15-PGDH, COX-2 and MVD. The Kaplan-Meier method was used to estimate the survival curves for each group, and the differences between the curves were analyzed according to the log-rank test. OS was measured from the date the surgery was carried out until mortality (from any cause) or the end of the observation period. The groups were classified by clinicopathological features, including gender (male vs. female), age (<70 vs. ≥70 years old), smoking history (never smoked vs. smoked), histological type (squamous cell carcinoma vs. adenocarcinoma), histological differentiation (poor vs. moderate or well), tumor size (≤3 vs. >3 cm), lymph node invasion (no invasion vs. invasion of ≥1 node) and TNM stage (I + II vs. III + IV). P<0.05 (two-sided) was considered to indicate a statistically significant difference. Statistical analyses were conducted using SPSS version 17.0 (SPSS, Inc., Chicago, IL, USA).

## Results

### Expression of 15-PGDH and COX-2 in the NSCLC and normal control group

**Figure 1:**
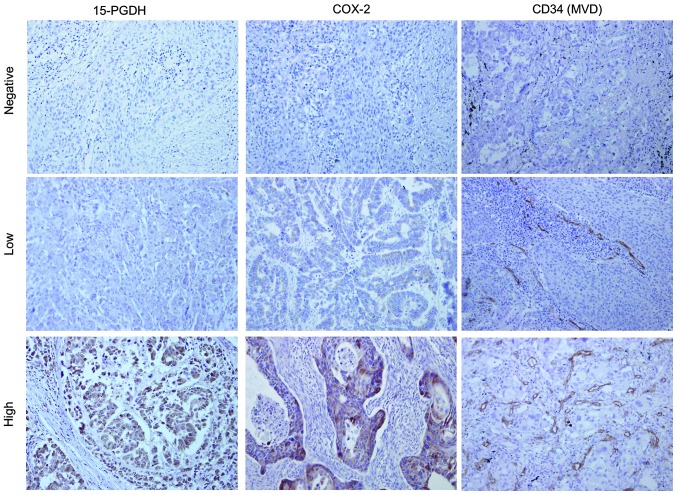
Immunohistochemical staining (magnification, ×200) of 15-PGDH, COX-2 and CD34 (MVD) in the NSCLC specimens are shown. Representative images of negative, low and high staining intensity, respectively. 15-PGDH, 15-hydroxyprostaglandin dehydrogenase; COX-2, cyclooxygenase-2; MVD, microvessel density.

The predominant pattern of 15-PGDH and COX-2 staining was a yellow or brownish-yellow stain in the cytoplasm. The different intensities of the staining are shown in Fig. 1. The high expression rate of 15-PGDH was 40% (14/35) in the NSCLC group and 100% (6/6) in the normal group (Table I), while the high expression rate of COX-2 was 65.7% (23/35) in NSCLC group and 0% (0/6) in normal group. The NSCLC tissue showed a significantly lower expression level of 15-PGDH (P=0.009) and a higher expression level of COX-2 (P=0.004) compared with normal lung tissue.

### Correlation of 15-PGDH, COX-2 and MVD with clinicopathological characteristics

Table II summarizes the association between 15-PGDH, COX-2 and MVD, and the clinicopathological parameters of NSCLC patients. There was no significant correlation between 15-PGDH and any of the assessed clinicopathological characteristics, including gender, age, smoking, histological type, histological differentiation, tumor size, lymph node invasion and TNM stage (P>0.05). In addition, COX-2 expression and MVD were not correlated with any clinicopathological characteristics (P>0.05).

### Correlation between 15-PGDH, COX-2 and MVD

**Figure 2:**
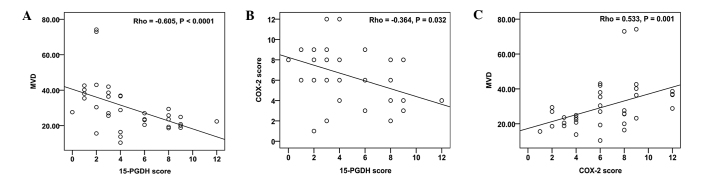
Correlation analysis between 15-PGDH, COX-2 and MVD. The expression of 15-PGDH was negatively correlated with (A) MVD and (B) COX-2 expression. (C) The expression of COX-2 was positively correlated with MVD. 15-PGDH, 15-hydroxyprostaglandin dehydrogenase; COX-2, cyclooxygenase-2; MVD, microvessel density.

The mean MVD for all 35 cases was 30.01 (range, 10.40–74.20). Representative immunohistochemical staining of CD34 (MVD staining) is shown in Fig. 1. The expression of 15-PGDH was negatively correlated with MVD (Fig. 2A; correlation coefficient (ρ) = −0.605, P<0.001) and COX-2 expression (Fig. 2B; ρ = −0.364, P=0.032). In addition, the MVD was significantly associated with COX-2 (Fig. 2C; ρ = 0.533, P=0.001).

### Survival analysis

**Figure 3:**
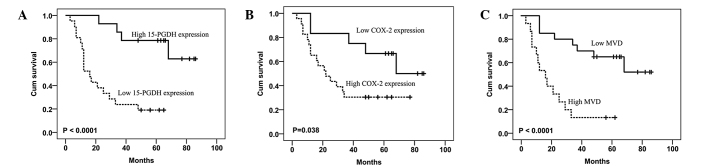
Kaplan-Meier analysis showed poorer overall survival of patients with (A) a low expression level of 15-PGDH, (B) a high expression level of COX-2 and (C) a low MVD. 15-PGDH, 15-hydroxyprostaglandin dehydrogenase; COX-2, cyclooxygenase-2; MVD, microvessel density.

The prognostic value of 15-PGDH, COX-2 and MVD on OS was evaluated in all NSCLC patients. The median survival time for the 35 patients was 34 months. The five-year OS rate for all patients was 42.9% (15/35). A strong prognostic impact of the three predictor variables on the survival curve was identified (Fig. 3). Patients with a low 15-PGDH expression level had a significantly poorer prognosis compared with patients with a high 15-PGDH expression level (Fig. 3A; log-rank, P<0.0001). Patients with a high COX-2 expression level (Fig. 3B) or a high MVD (Fig. 3C) had a shorter survival time (log-rank, P=0.038 and P<0.0001, respectively).

## Discussion

15-PGDH is regarded as a tumor suppressor, and has been shown to be overexpressed in cancer cells, including breast, colon, lung and glioma cells, resulting in reduced cellular proliferation (4). Furthermore, mice inoculated with cancer cells that were transfected with 15-PGDH showed a marked retardation of xenograft tumor growth (3,4). The present study found that the expression of 15-PGDH was low in the human NSCLC tissues compared with the normal lung tissues, which is consistent with the downregulation of 15-PGDH in numerous human cancer specimens, including those of lung (19), colorectal (20,21), gastric (10,22), breast (7), bladder (11) and pancreatic (8) cancers. These findings indicate that the reduction of 15-PGDH may be associated with the occurrence of NSCLC. Furthermore, it was identified that there was no significant association between the expression of 15-PGDH and tumor differentiation, node invasion and TNM stage. Lim *et al* (21) reported similar data in colorectal cancer. However, a loss of 15-PGDH has been positively correlated with the differentiation, distant metastasis and TNM stage of gastric cancer (10,22). These discrepancies are likely affected by intratumoral heterogeneity and the sample size. The loss of 15-PGDH may have a role in the occurrence, but not the progression of lung cancer.

15-PGDH is known to be an endogenous COX-2 antagonist (23), and the increased expression level of COX-2 has been observed to be inversely associated with the expression of 15-PGDH in various cancer tissues (18,22,24–27). Based on these studies, the association of 15-PGDH and COX-2 was investigated in the present study. The expression of 15-PGDH was shown to be negatively correlated with the expression of COX-2 in the human NSCLC specimens. This result supports the hypothesis that 15-PGDH and COX-2 are reciprocally regulated in cancer. In the present study, overexpression of COX-2 and low levels of 15-PGDH were simultaneously detected in the lung cancer tissues. Stimulation of A549 human lung adenocarcinoma cells with IL-1β revealed an increase in COX-2 expression and endogenous synthesis of PGE2, accompanied by the downregulation of 15-PGDH (24). Overexpression of COX-2 and repression of 15-PGDH may coordinately increase the level of PGE2 in the tumor microenvironment and exacerbate the carcinogenic process (24).

Angiogenesis is important for cancer progression and prognosis (28). MVD, as a standard quantification of tumor angiogenesis, is considered to be a prognostic indicator of solid tumors (29–32), and high levels of MVD indicate a short survival time in lung cancer patients (33). The data from the present study are consistent with these results. Previous studies have demonstrated that COX-2-mediated PGE2 promotes neovascularization through the upregulation of pro-angiogenic factors, such as vascular endothelial growth factor (34,35). However, few studies have reported the function of 15-PGDH in tumor angiogenesis. Huang *et al* (12) observed that the MVD was significantly reduced in xenografts derived from 15-PGDH-overexpressing H358 lung cancer cells, and that the expression of 15-PGDH could inhibit capillary tube formation of human umbilical endothelial cells and VEGF production. Consistent with these results, the present study showed that the expression of 15-PGDH was inversely associated with MVD in human lung cancer tissues. Additionally, it has been reported that apoptotic cells, which originated from stressed or damaged tissues, were found to induce PGE2-modulated angiogenic properties in human macrophages, accompanied by COX-2 upregulation and repression of 15-PGDH (36). A previous study showed that the combinatory treatment of 15-PGDH gene therapy and COX-2 inhibitor significantly inhibits murine breast tumor growth and tumor angiogenesis compared with monotherapy or controls (37). The effect of this combination treatment was associated with a significant reduction of PGE2 in the serum, which resulted from increased 15-PGDH and decreased COX-2 expression in tumor tissues (37). An anti-angiogenic mechanism may contribute to the tumor suppressor function of 15-PGDH, and it has been shown that administration with a COX-2 inhibitor enhances the efficacy of 15-PGDH (12,37). When considering the relevance of 15-PGDH and COX-2 to neovascularization, the present study found that the low 15-PGDH and high COX-2 expression levels were associated with an unfavorable prognosis in the NSCLC patients. These findings indicate that 15-PGDH and COX-2 may affect prognosis by regulating the angiogenic activity of lung cancer cells.

Although the present study demonstrated that 15-PGDH and COX-2 reciprocally regulated angiogenesis and were prognostic predictive factors of patients with NSCLC, there are several limitations to consider. Firstly, this study had a relatively small sample size, which could affect the statistical significance. Furthermore, limited to the small number of patients, a multivariate survival analysis could not be conducted to determine the independent prognostic predictors. Thirdly, the present study only included two main histological types of NSCLC specimens. Therefore, the data regarding the expression of markers may not reflect the comprehensive status of NSCLC. These results may only apply to squamous cell carcinoma and adenocarcinoma rather than to all NSCLC histological types.

In conclusion, the present respective study found downregulated 15-PGDH expression in human NSCLC tissues. The correlations between 15-PGDH and COX-2 expression, and their association with angiogenesis and prognosis, was evaluated. To the best of our knowledge, this is the first study to determine that a loss of 15-PGDH is associated with a poor prognosis in patients with NSCLC. These results provide preliminary evidence for further studies in NSCLC, including those with a larger sample size, prospective clinical studies and those with therapies targeting 15-PGDH and COX-2, such as the use of COX-2 inhibitors.

## Figures and Tables

**Table I:** Expression of 15-PGDH and COX-2 in NSCLC tissues and matched normal lung tissues.

	15-PGDH expression			COX-2 expression		
				
Groups	High, n (%)	Low, n (%)	P-value	High, n (%)	Low, n (%)	P-value
NSCLC tissue (n=35)	14 (40.0)	21 (60.0)	0.009^a^	23 (65.7)	12 (34.3)	0.004^a^
Normal lung tissue (n=6)	6 (100.0)	0 (0.0)		0 (0.0)	6 (100.0)	

a P<0.05.

15-PGDH, 15-hydroxyprostaglandin dehydrogenase; COX-2, cyclooxygenase-2; NSCLC, non-small cell lung cancer.

**Table II:** Correlation between 15-PGDH, COX-2, MVD and clinicopathological features.

		15-PGDH expression			COX-2 expression			MVD	
				
Characteristics	No.	High, n	Low, n	P-value	High, n	Low, n	P-value	Mean ± SD	P-value
Patients included	35	14	21		23	12		30.01±13.97	
Gender				0.392			1.000		0.246
Male	22	10	12		14	8		32.14±15.65	
Female	13	4	9		9	4		26.40±10.08	
Age, years				1.000			0.918		0.475
<70	15	6	9		10	5		31.99±13.74	
≥70	20	8	12		13	7		28.52±14.31	
Smoking				0.324			0.827		0.655
No	14	7	7		10	4		31.33±14.04	
Yes	21	7	14		13	8		29.13±14.20	
Histological type				0.486			0.537		0.806
Squamous cell carcinoma	20	7	13		14	6		29.50±13.87	
Adenocarcinoma	15	7	8		9	6		30.69±14.57	
Histological differentiation				1.000			0.576		0.579
Moderate/Well	24	10	14		17	7		30.92±15.24	
Poor	11	4	7		6	5		28.04±11.10	
Tumor size				0.056			1.000		0.969
≤3 cm	10	7	3		7	3		29.86±16.32	
>3 cm	25	7	18		16	9		30.07±13.29	
Lymph node invasion				0.407			0.903		0.518
No	17	8	9		11	6		28.41±15.12	
Yes	18	6	12		12	6		31.52±13.04	
TNM stage				0.158			0.329		0.532
I+II	24	12	12		14	10		30.51±15.40	
III+IV	11	2	9		9	2		28.92±10.79	

15-PGDH, 15-hydroxyprostaglandin dehydrogenase; COX-2, cyclooxygenase-2; MVD, microvessel density; TNM, tumor node metastasis; SD, standard deviation.
